# Identifying patients with an unfavorable prognosis in early stages of colorectal carcinoma

**DOI:** 10.18632/oncotarget.25384

**Published:** 2018-06-08

**Authors:** Alexander Hendricks, Greta-Lou Eggebrecht, Alexander Bernsmeier, Reinhild Geisen, Katharina Dall, Anna Trauzold, Thomas Becker, Holger Kalthoff, Clemens Schafmayer, Christian Röder, Sebastian Hinz

**Affiliations:** ^1^ Department of General and Thoracic Surgery, University Hospital Schleswig-Holstein, Campus Kiel, Kiel, Germany; ^2^ Institute for Experimental Cancer Research, Christian-Albrechts University, Kiel, Germany

**Keywords:** colorectal cancer, liquid biopsy, circulating tumor cells, cytokeratin 20, prognostic marker

## Abstract

**Background:**

In recent years, the concept of liquid biopsy diagnostics in detection and progress monitoring of malignant diseases gained significant awareness. We here report on a semi-quantitative real-time cytokeratin 20 RT-PCR-based assay, for detecting circulating tumor cells within a fraction of peripheral blood mononuclear cells in colorectal cancer patients.

**Methods:**

In total, 381 patients were included. Prior to surgical tumor resection, a peripheral blood sample was drawn. Mononuclear cells were isolated by Ficoll centrifugation and a cytokeratin 20 qRT-PCR assay was performed. Quantitative PCR data was assessed regarding histopathological characteristics and patients´ clinical outcome.

**Results:**

A cut-off value was determined at ≥ 2.77 [EU]. Stratifying patients by this cut-off, it represents a statistically highly significant prognostic marker for both the overall and disease-free survival in the entire cohort UICC I-IV (both p<0.001) and in early tumor stages UICC I+II (overall survival p=0.003 and disease-free survival p=0.005). In multivariate analysis, the cut-off value stands for an independent predictor of significantly worse overall and disease-free survival (p=0.035 and p=0.047, respectively).

**Conclusion:**

We successfully established a highly sensitive real-time qRT-PCR assay by which we are able to identify colorectal cancer patients at risk for an unfavorable prognosis in UICC I and II stages.

## INTRODUCTION

Colorectal cancer (CRC) still counts for the second most frequent cause of cancer death [1]. Around 90% of deaths are owed to formation of distant metastases mostly in liver and lung. In patients with UICC (Union internationale contre le cancer) stage III and IV CRC, a significant increase in survival could be achieved in recent decades, primarily owed to new therapeutic regimes including antibody-based immunotherapies [2, 3]. This progress though was not fully transferred to patients with early stage CRC [4]. According to current guidelines, adjuvant therapy in stage II CRC is only administered if clinical risk factors (e.g. tumor perforation, pT4 tumor, lymph vessel invasion) are apparent. The development of markers that provide additional prognostic information and also identify patients at risk for future metastases, are urgently needed [5].

The presence of circulating tumor cells (CTC) in the peripheral blood has been shown to identify CRC patients with an unfavorable prognosis [6–9]. To date, various techniques for CTC detection have been presented [10, 11]. Previously, we established a qualitative nested endpoint RT-PCR specific for cytokeratin (CK)20-mRNA, coding for an intermediate filament protein of epithelial cells, which has been shown to detect CTC within a fraction of peripheral blood mononuclear cells (PBMC) with a high specificity and sensitivity in the blood of CRC patients [12, 13]. Thus, CK20 is a broadly accepted biomarker for the detection of CTC in patients suffering from CRC [6, 12–15].

In this prospective study, we report on a refined quantitative real-time CK20 RT-PCR, that bears the possibility to semi-quantitatively analyze the CTC/PBMC fraction in the peripheral blood. This method allows to increase the sensitivity of detection and define a cut-off value, which identifies, even in early tumor stages, CRC patients with a bad prognosis. Furthermore, to maximize the analysis´ sensitivity, we established a dual-marker qRT-PCR analyzing ectopic CK20- and epithelial growth factor receptor (EGFR)-mRNA expression in Ficoll-enriched PBMC-fractions from peripheral blood. EGFR plays a significant role in CRC [16, 17]. Its level of expression greatly increases with histopathologically advanced tumor growth [18] and it is linked to a significantly worse overall survival (OS) in CRC patients [19].

To our best knowledge, this is the first study showing a negative prognostic role of CTC within a PBMC fraction detected by a real-time qRT-PCR against CK20 in CRC patients in a large representative cohort. We were able to identify an additional molecular risk factor for CRC patients with UICC stages I and II to stratify patients who might benefit from adjuvant chemotherapy.

## RESULTS

### Patient and clinical characteristics

The study cohort consisted of 381 patients, all diagnosed with histologically confirmed colorectal cancer. A synopsis of the clinical data is given in Table 1. 224 patients were diagnosed with colon cancer and 157 with a rectal carcinoma. The mean age at the time of surgery was 68.5 years (range: 32 – 95 years). The median follow-up was 34 months (range: 0 – 151 months) and the median overall survival (OS) was 24 months (range: 0 – 118 months).

**Table 1:** Patient demographics, clinical characteristics and univariate analysis (log rank test) influencing the 5-year overall survival (OS) and 5-year disease free survival (DFS)

	*N* (%)	5y-OS [%]	univariate analysis (*P*)	5y-DFS [%]	univariate analysis (*P*)
**All**	381 (100.0)	67.5		58.8	
**age [years]**					
< 70	210 (55.1)	58.7	**0.038**	55.6	0.185
≥ 70	171 (44.9)	54.7		51.6	
**Sex**					
male	235 (61.7)	54.5	0.935	52.4	0.755
female	146 (38.3)	62.0		57.1	
**tumour site**					
colon	224 (58.8)	63.9	0.083	58	0.071
rectum	157 (41.2)	49.0		48	
**UICC Stage**					
I	118 (31.0)	87.9	**<0.001**	86.8	**<0.001**
II	91 (23.9)	74.6		67.2	
III	87 (22.8)	53.5		47.7	
IV	85 (22.3)	5.6		3.9	
**pT**					
T0	8 (2.1)	58.3	**<0.001**	58.3	**<0.001**
T1	40 (10.5)	90.4		90.9	
T2	100 (26.2)	83.6		79.0	
T3	186 (48.8)	42.7		39.9	
T4	47 (12.3)	25.9		19.3	
**pN**					
N0	225 (59.1)	76.9	**<0.001**	73.5	**<0.001**
N1	82 (21.5)	43.5		41.6	
N2	74 (19.4)	16.5		10.2	
**pM**					
M0	296 (77.7)	74.0	**<0.001**	69.9	**<0.001**
M1	85 (22.3)	5.5		3.9	
**neoadjuvant treatment**					
yes	54 (14.3)	64.2	0.198	57.2	0.535
no	323 (84.6)	55.7		53.4	
unknown	4 (1.1)				
**adjuvant treatment**					
yes	136 (35.7)	44.1	**0.001**	38.8	**<0.001**
no	237 (62.2)	65.5		62.9	
unknown	8 (2.1)				
**CK20 expression [EU]**					
< 2.77	220 (57.7)	69.6	**<0.001**	66.2	**<0.001**
≥ 2.77	161 (42.3)	39.8		37.6	
**EGFR expression**					
positive	171 (44.9)	57.0	0.979	52.7	0.880
negative	210 (55.1)	56.8		55.0	

All *P* values in bold, are regarded as statistically significant. CTC: circulating tumour cells; CK20: cytokeratin 20; EGFR: epidermal growth factor receptor; EU: expression units.

### Clinicopathogical characteristics, CTC detection and prognosis

The 5-year OS and DFS rate for all patients in this study was 67.5% and 58.8%, respectively. As expected, advanced tumor stages correlated with worse patients´ outcome (Supplementary Figure 1).

The overall detection rate of CK20-positivity by qRT-PCR was 53.0% (202/381 patients) and 44.9% (171/381 patients) for EGFR-positivity. All experimentally derived qPCR data is shown in detail in Supplementary Table 1. Detection of CK20 alone was highly significantly correlated with a poor prognosis in univariate analysis (OS and DFS, both *P*<0.001), whereas the detection of EGFR alone did not reveal any significant correlation with the OS (*P*=0.979 and DFS (*P*=0.880) (data not shown). Furthermore, the dual-marker analysis of both, CK20 and EGFR did not lead to an increase in predictive sensitivity of the patients´ outcome. Likewise, the detection of EGFR-positivity in CK20-negative patients did not show any correlation with the OS or DFS rate (data not shown).

### Control group and sensitivity analysis by spiking experiments

By applying the qRT-PCR assay to blood samples of the control cohort of healthy donors, the specificity of the assay was determined. None of the 15 tested subjects were positive for either CK20 or EGFR. By serial dilution of live HT29 tumor cells into blood, the sensitivity of the assay was optimized up to the detection of 1 cell per 1 ml whole blood (Supplementary Figure 2).

### ROC-curve analysis of CK20 expression levels defined a diagnostic cut-off threshold

The aim was to utilize the quantitative expression levels of CK20 mRNA, to serve as a prognostic marker in predicting the course of disease. By applying ROC-curve analysis, the quality of testing for CK20 mRNA expression was distinctively confirmed (Supplementary Figure 3). Reasoning these results, high expression levels stand for a significantly worse outcome. In this analysis, a strong cut-off value of 2.77 relative mRNA expression units was determined by the Youden´s index.

**Figure 1:**
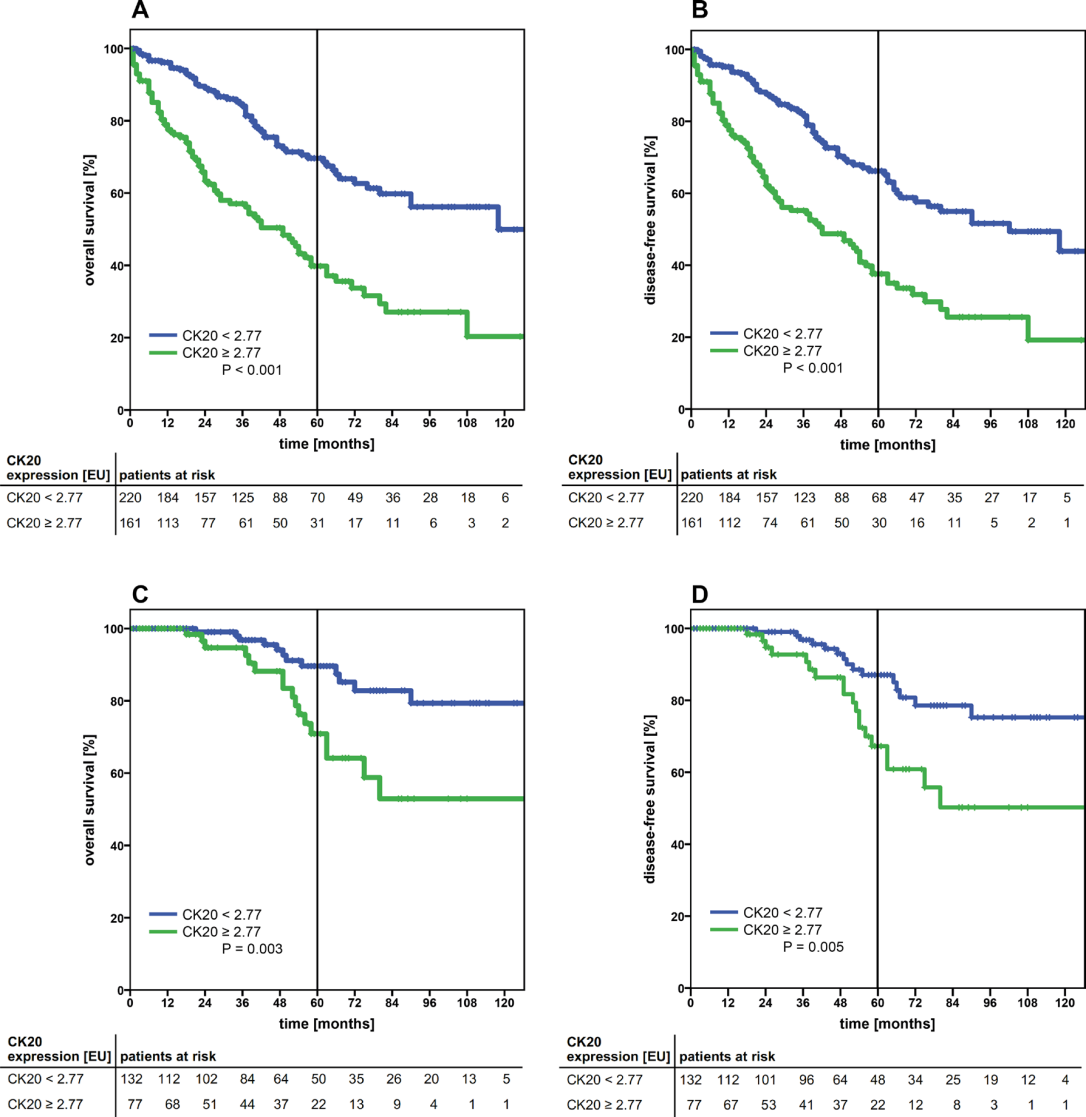
Kaplan-Meier survival analysis of the cumulative overall survival **(A, C)** and disease-free survival **(B, D)** of patients with colorectal carcinoma of UICC stage I-IV (A, B) and UICC stage I+II (C, D) according to the cytokeratin-20 mRNA expression levels (high, ≥ 2.77 EU; low, < 2.77 EU). The tables under each plot show the number of patients at risk at each time point in the graph. The 5-year survival is indicated by thick vertical lines. P-values were calculated by log-rank tests. CK20: cytokeratin 20; EU: expression units.

Adopting the cut-off value to the outcome of the entire cohort, patients with high CK20 gene expression (≥ 2.77) showed a significantly worse outcome (*P*<0.001 in both the OS and DFS) (Figure 1A and 1B). Patients with low CK20 gene expression (< 2.77) had a 5-year OS of 69.6%, whereas in the cohort of patients with high CK20 gene expression (≥ 2.77) the 5-year OS dropped to 39.8%. Similar results were observed for the DFS (Table 1). Analyzing the subgroups of colon and rectal carcinoma independently, applying the cut-off for CK20 expression, both subgroups showed significant correlation with a worse OS and DFS (both *P*<0.001, Supplementary Figure 4). Higher tumor stages (UICC IV) and locally advanced tumor growth (pT4), coincided with higher CK20 mRNA expression levels and significantly more often the cut-off value was exceeded (*P*<0.001 and *P*=0.004, respectively) (Table 2). Interestingly, local lymph node metastasis as a sign of locally progressive tumor growth did not correlate with higher CK20 mRNA expression levels. Though, the data suggests a clinically relevant trend (Table 2).

**Table 2:** Correlation of quantitative detection of CK20-mRNA and association to clinical characteristics determined by χ^2^ testing

	CK20 ≥2.77 EU *N* (%)	*P*
**all**	161 (42.3)	
**age [years]**		
< 70	82 (39.0)	0.097
≥ 70	79 (46.2)	
**Sex**		
male	92 (39.1)	0.073
female	69 (47.3)	
**tumour site**		
colon	91 (40.6)	0.253
rectum	70 (44.6)	
**UICC stage**		
I	42 (35.6)	**<0.001**
II	35 (38.5)	
III	26 (29.9)	
IV	58 (68.2)	
**pT**		
T0	4 (50.0)	**0.004**
T1	8 (20.0)	
T2	38 (38.0)	
T3	83 (44.6)	
T4	28 (59.6)	
**pN**		
N0	88 (39.1)	0.083
N+	73 (46.8)	
**pM**		
M0	103 (34.8)	**<0.001**
M1	58 (68.2)	
**neoadjuvant treatment**		
yes	24 (44.4)	0.429
no	137 (42.1)	
**adjuvant treatment**		
yes	57 (41.9)	0.460
no	104 (43.0)	

All *P* values in bold are regarded as statistically significant; CK20: cytokeratin 20; EU: expression units.

Applying a multivariate analysis for all variables showing a significant correlation to survival in the univariate analysis, we could prove that CK20 mRNA expression above or below the cut-off in CRC patients represents an independent prognostic marker in the entire cohort (UICC stages I-IV) for the OS (HR 2.49; 95% CI 1.77 – 3.49; *P*<0.001) and DFS (HR 2.34; 95% CI 1.69 – 3.22; *P*<0.001) (Table 3, upper panel). Moreover, also the UICC staging was significantly proven as an independent prognostic factor in both, OS and DFS (HR 7.85; 95% CI 5.08 – 12.15; *P*<0.001 and HR 7.39; 95% CI 4.90 – 11.16; *P*<0.001, respectively). The other variables tested (age and adjuvant treatment) turned out to be not correlated independently (Table 3, upper panel).

**Table 3:** Multivariate Cox regression analysis and hazard models of independent factors influencing overall- and disease-free survival in the entire study cohort (UICC I-IV) and early tumour stages (UICC I+II)

	overall survival		disease-free survival	
	multivariate HR (95% CI)	*P*	multivariate HR (95% CI)	*P*
**UICC I-IV**				
CK20 < 2.77 vs ≥ 2.77 [EU]	2.49 (1.77 – 3.49)	**<0.001**	2.34 (1.69 – 3.22)	**<0.001**
age < 70 vs. ≥ 70 [years]	1.27 (0.90 – 1,79)	0.172	n.d.	
**UICC I+II vs. III+IV**	7.85 (5.08 – 12.15)	**<0.001**	7.39 (4.90 – 11.16)	**<0.001**
adj. treatment yes vs. no	1.10 (0.77 – 1.56)	0.610	1.09 (0.78 – 1.53)	0.606
**UICC I+II**				
CK20 < 2.77 vs. ≥ 2.77 [EU]	2.25 (1.06 – 4.77)	**0.035**	2.01 (1.01 – 4.01)	**0.047**
age < 70 vs. ≥ 70 [years]	1.59 (0.73 – 3.47)	0.245	n.d.	
colon vs. rectum	0.22 (0.09 – 0.50)	**<0.001**	0.27 (0.13 – 0.57)	**0.001**
pT1/2 vs. pT3/4	4.50 (1.99 – 10.17)	**<0.001**	4.17 (1.97 – 8.85)	**<0.001**

All *P* values in bold, are regarded as statistically significant; n.d.: the value was not significant in univariate analysis and therefore not considered in multivariate analysis; HR: hazard ratio, CI: confidence interval; CK20: cytokeratin 20; EU: expression units.

### Subgroup analysis of UICC I+II, II+III and III+IV patients

Since usually only patients with advanced disease (UICC stages III and IV) receive adjuvant therapy according to the treatment guidelines, a stratification of the study cohort is clinically particularly interesting. To determine the role of CK20-expression as a negative prognostic marker in early tumor stages, the cohort was stratified with respect to early tumor stages (I + II) only. Within this cohort, high mRNA Expression levels of CK20 (≥ 2.77) were a highly significant marker for worse OS and DFS (*P*=0.003 and p=0.005, respectively) (Figure 1C+1D and Table 4). Furthermore tumor localization in colon vs. rectum, pT category and patients´ age emerged as parameters with significant correlation to the OS and DFS (Table 4).

**Table 4:** Description and analysis (log-rank test) of factors influencing the 5-year overall survival (OS) and 5-year disease free survival (DFS) rate in the subgroup of UICC I+II patients

	*N* (%)	5y-OS [%]	univariate analysis (*P*)	5y-DFS [%]	univariate analysis (*P*)
**all**	209 (100.0)	92.9		89.4	
**age [years]**					
< 70	115 (55.0)	83.4	**0.048**	81.3	0.062
≥ 70	94 (45.0)	81.7		77.3	
**Sex**					
male	135 (64.6)	79.2	0.276	75.9	0.372
female	74 (35.4)	89.9		87.6	
**tumour site**					
colon	123 (58.9)	92.0	**0.010**	87.5	**0.019**
rectum	86 (41.1)	72.3		71.0	
**pT**					
1	37 (17.7)	94.4	**0.005**	89.3	**0.007**
2	80 (38.3)	97.3		87.2	
3	81 (38.8)	74.8		71.5	
4	11 (5.3)	50.0		50.0	
**CK20 expression EU**					
< 2.77	132 (63.2)	89.6	**0.003**	87.1	**0.005**
≥ 2.77	77 (36.8)	70.9		67.3	

All *P* values in bold, are regarded as statistically significant. CK20: cytokeratin 20; EU: expression units.

These parameters were also explored in a multivariate analysis, which demonstrated that the CK20 expression level remains significant as an independent prognostic marker for a worse OS (HR 2.25; 95% CI 1.06 – 4.77; *P*=0.035) and DFS (HR 2.01; 95% CI 1.01 – 4.01; *P*=0.047) (Table 3, lower panel). Further, the other variables tested in univariate analysis, tumor localization, pT-category also prove to be highly significant independent variables in predicting the patients´ outcome, whereas patients age was not proven to be an independent predictor (Table 3, lower panel).

Analyzing the subgroup of patients with locally advanced and metastatic CRC (UICC III+IV) we were also able to prove the expression of CK20 mRNA being a significant prognostic marker for both, the OS and DFS (both *P*<0.001) (data not shown).

**Figure 2:**
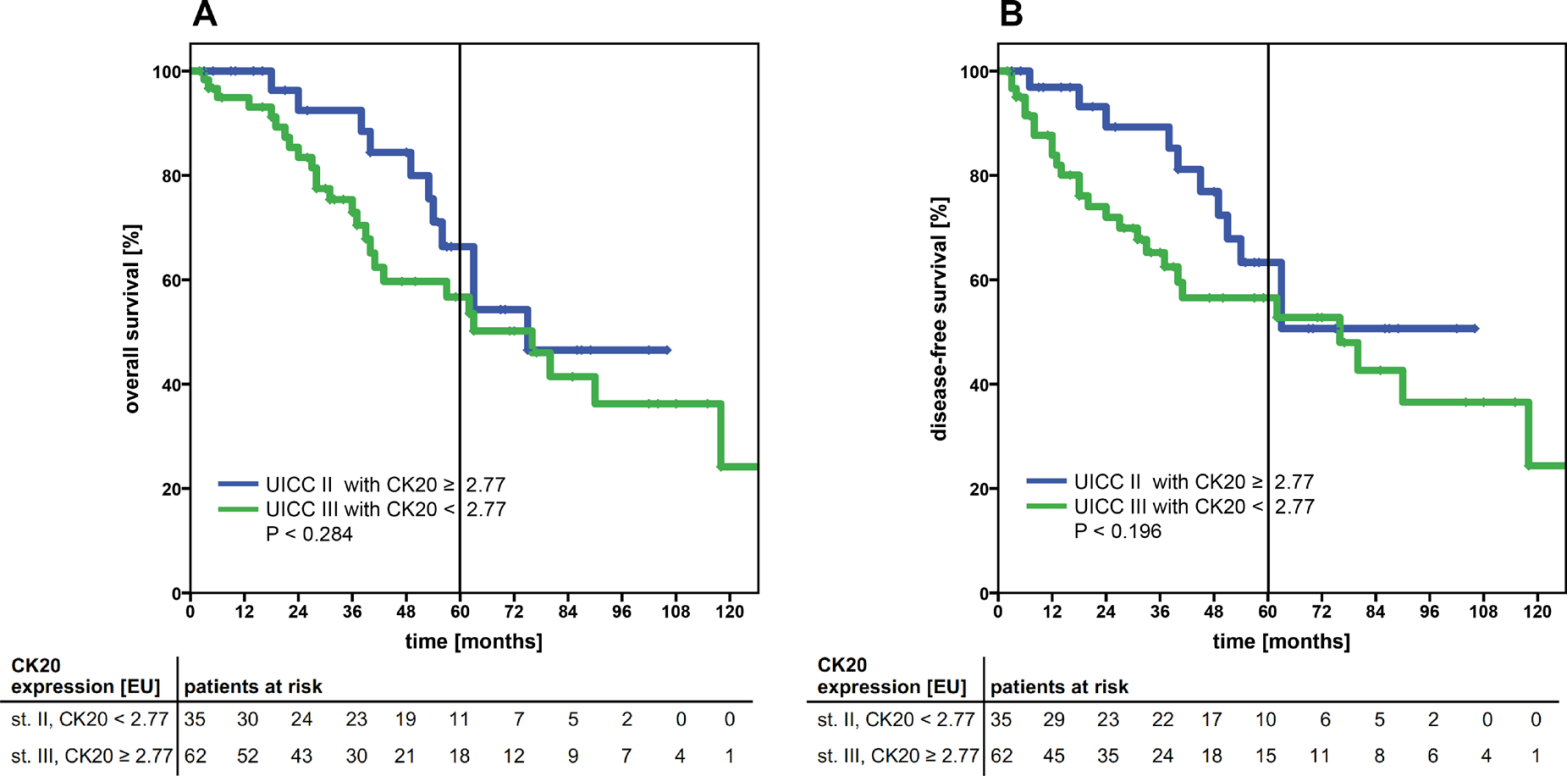
Kaplan-Meier survival analysis of the cumulative overall survival **(A)** and disease-free survival **(B)** of patients with UICC stage II and high CK20 mRNA expression levels (≥ 2.77 EU cut-off) compared to UICC stage III patients with low CK20 expression levels (< 2.77 EU). Both patient sub-cohorts showed a comparable cumulative survival. The tables under each plot show the number of patients at risk at each time point in the graph. The 5-year survival is indicated by thick vertical lines. P-values were calculated by log-rank tests. CK20: cytokeratin 20; EU: expression units.

Another clinically highly interesting issue is the problem of over- or under treatment of cancer patients. According to the medical guidelines, the majority of patients diagnosed with UICC II CRC are not admitted to an adjuvant treatment, whereas patients with stage III CRC are. Therefore, we explored the subgroup of UICC II and III patients and stratified these in potential patients at risk. Patients staged UICC II with high CTC CK20 gene expression (≥ 2.77) (patients at risk, possibly being undertreated) were correlated to patients staged UICC III with low CTC CK20 gene expression (< 2.77) (patients possibly excessively treated). Interestingly, no statistical difference in the OS or DFS (*P*=0.284 and *P*=0.196, respectively) was observed (Figure 2A+2B), suggesting a further possible clinical impact of applying a cut-off value for quantitative CK20-expression detection.

## DISCUSSION

At all, the expression of CK20 allows for a review of cancer recurrence and therapeutic efficiency, as well as prognosis in colorectal cancer patients. In future, it may even serve as liquid biopsies.

In this prospective study with a large and representative cohort of CRC patients, we analyzed the prognostic relevance of CK20 expression of a PBMC fraction containing CTC and patients´ clinical outcome. We proved sole detection of CK20 expression to be a highly significant independent marker for OS and DFS in CRC. Further, we demonstrated proof of concept for our semi-quantitative real-time CK20 RT-PCR and could process the clinically most interesting subgroup of UICC I and II patients in more depth. We were able to identify patients at risk in these early stages precisely by sole detection of CK20 expression and further by defining a clinically relevant cut-off value of quantitative CK20 expression in this cohort.

Due to its anticipated clinical relevance in oncological diagnostics and disease monitoring, the concept of liquid biopsy diagnostics for solid tumors has been emphasized considerably in the recent literature [11, 20]. The biological basis for liquid biopsy analysis lies in the various molecular and/or cellular traces of a solid tumor in the blood as circulating cell-free tumor DNA (cfDNA), miRNAs, exosomes, proteins and CTC [21] among other tumor-derived biomarkers. Recently arising is the (experimentally-based) hypothesis of CTC being highly heterogeneous, comprising epithelial tumor cells, tumor cells after epithelial-to-mesenchymal transition (EMT), and circulating tumor stem cells (CTSC) [22, 23]. Hence, various biomarkers and techniques for detecting CTC have been implemented [24]. In particular though, the transmembrane glycoprotein EpCAM as a general endodermal epithelial cell marker is of broad interest. The up to now exclusively FDA-approved immunomagnetic anti-EpCAM assay, employed by the CellSearch System^©^, is utilized by many research groups. Yet, a growing number of studies show detection rates of CTC in CRC patients to be modest with this system [25, 26].

During the process of metastasizing, some cells undergo EMT and epithelial markers such as EpCAM are either lost, or significantly downregulated [27]. Instead, upregulation of mesenchymal markers such as vimentin are seen. This mesenchymal cell fraction is said to be considerably more hostile with a more aggressive phenotype and an increased metastatic potential in CRC patients [28–30]. Tests designed for detection of these markers, therefore lack precision - the real load of CTC may be underestimated by the anti-EpCAM assays [31–33].

Up to now, to the best of our knowledge, many publications focused on the overall predictive value of CTC in CRC patients and numerous studies comprised cohorts of patients with a high tumor burden of even metastatic colorectal cancer patients. Cohort numbers were remote and only few studies targeted clinically relevant histopathological subsets in terms of UICC staging.

CRC patients with stage III and IV disease experienced a substantial increase in disease-free survival in recent decades. New therapeutic regimes had been introduced in particular addressing metastatic CRC patients. At the same time the OS of patients suffering from early-stage tumors had been improved sparsely. In our cohort, the 5-year OS in patients with limited disease (no regional lymph node metastasis) was 85.3%. Hence, still a significant number of CRC patients die due to tumor burden and later development of distant metastasis, whereby the initial extend of tumor load is sparse. Desirable would be to establish a protocol to identify these patients at risk in early stage disease and to discriminate this clinically highly relevant sub-cohort further. In 2015, Bork et al. [34] investigated the clinical relevance of CTC by applying the Cell-Search system in a large cohort of CRC patients. Patients with less tumor burden (UICC I-III) were analyzed independently regarding the CTC count and prognostic value. They proved the predictive importance of CTC in early tumor stages. Contrary to these findings, Sotelo *et al*. [35] published their results in 2015, stating the CTC count not to have any prognostic impact in stage III colorectal cancer patients. Likewise, detection was carried out by the CellSearch^©^ system. As discussed beforehand, the CTC detection by the EpCAM reliant CellSearch^©^ method is arguably inferior. In their study, Iiunuma *et al*. [36] demonstrated the CTC detection by PCR (CK+/CEA+/CD133+) in a large cohort of 420 CRC patients to be significantly superior relative to the CellSearch^©^ system.

In our study, we now applied a refined quantitative real-time CK20- and EGFR-specific RT-qPCR. The overall detection rate of 53% for CK20 expression was significantly higher than with the up to now utilized nested RT-PCR in our work group [6, 13]. In a representative cohort of CRC patients, the detection of CK20 expression within a CTC containing subset of PBMC presents a highly significant predictive marker in the prognosis of patients. Detection of CK20 expression by qRT-PCR has the ability of independently acting as a liquid biopsy. High detection rates of CK20 expression in early tumor stages may be arguable, but by defining a cut-off value in the cohort of CRC patients it is possible to identify patients who might be at risk and may experience a worse outcome.

Our data evidently shows that CRC patients with high CK20 expression have a significantly worse OS and DFS. Furthermore, we show that by applying a cut-off value, it is possible to identify patients at risk even in UICC I and II stages that might benefit form additional adjuvant treatment.

## MATERIALS AND METHODS

### Patient cohort and study design

All 381 patients included underwent complete oncological resection (R0) for a histologically verified colorectal carcinoma between the years 2004 and 2013 in the Department of General Surgery and Thoracic Surgery, University Hospital Schleswig Holstein, Campus Kiel. Patients with stage III or IV colon cancer were recommended to receive adjuvant or palliative chemotherapy, respectively, according to the therapy guidelines. In case of synchronous liver metastases, the patients underwent resection of the primary and the liver metastases in one operation. Patients with rectal carcinoma staged higher than uT3 or uN1 underwent neoadjuvant radio-chemotherapy with 50.4 Gy and two cycles of chemotherapy with 5-fluorouracil (5-FU) followed by 4 cycles of chemotherapy with 5-FU after surgery (according to [37]). In some cases, this regimen deviated according to the consensus meeting of the interdisciplinary tumor board.

The study was approved by the local ethics committee of the Medical Faculty, Christian-Albrechts University Kiel (reference no. A110/99). All patients gave written informed consent before inclusion to the study. Classification of the pathological tumor stage was handled by the Department of Pathology, University Hospital Schleswig Holstein, Campus Kiel, according to the TNM-classification. Clinical data was obtained from the clinical research database of the oncological biobank BMB-CCC of the Comprehensive Cancer Center Kiel and data was verified by re-examination of original patient records. Follow-up data was surveyed in cooperation with general practitioners and with the Cancer Registry of the Federal State of Schleswig-Holstein (Bad Segeberg, Germany). Clinical and follow-up data were then analyzed relating to the degree of CK20 and EGFR mRNA expression detected by the qRT-PCR. In case of CK20 positivity, the level of marker expression was calculated and included into the analysis. Qualitative and quantitative data were used to stratify patients at risk and the prognostic relevance of CK20- or EGFR-expression in the blood samples of CRC patients was analyzed.

### Control group

The control cohort consisted of 15 healthy volunteers. Peripheral blood samples were taken and analyzed as described in the following. Written consent for participating in this study was acquired prior to blood drawing. Investigation of the samples was likewise covered by the approval of the local ethics committee as described before.

### Liquid biopsy collection and isolation of blood mononuclear cell fractions

Instantly prior to surgery, a blood sample was drawn from a central venous line into a lithium heparin-Monovette (Sarstedt, Nümbrecht, Germany). All samples were kept at room temperature (18°C-25°C) and were further processed within 0.5-2 hours. Separation of the mononuclear cell (MNC) fraction was performed by centrifugation through a Ficoll-Hypaque density cushion (GE Healthcare, Freiburg, Germany). MNCs were then isolated, washed in PBS and counted.

### Isolation of total RNA and cDNA synthesis

MNCs were subsequently lysed with RNAPure™ reagent (VWR Peqlab, Darmstadt, Germany) and total RNA preparation was carried out according to the manufacturer's protocol. RNA concentration was measured by a NanoDrop 2000c Spectrophotometer (VWR Peqlab). RNA integrity was verified using a Bioanalyzer 2100 instrument (Agilent Technologies, Böblingen, Germany).

cDNA was obtained by reverse transcription of 3 μg total RNA (Maxima First Strand cDNA Synthesis Kit, Thermo Fisher Scientific, Darmstadt, Germany) according to the manufacturer's protocol.

### Realtime-qPCR and analysis

Realtime qPCR was conducted using TaqMan gene expression assays and the TaqMan Universal Master Mix II (Life Technologies, Darmstadt, Germany) with 200 ng cDNA template on a StepOnePlus instrument (Life Technologies). Assays were run in total volumes of 20 μl on 96-well plates (Sarstedt) and the following TaqMan gene expression assays were used: KRT20 (CK20), Hs00966063_m1; EGFR Hs01076078_m1; TBP, Hs00427621_m1. All samples were run in triplicate. The mean threshold cycles of triplicate reactions were computed using the StepOne software v. 2.1 (Life Technologies) after adjustment to the same threshold of all runs for each TaqMan assay on different plates. Gene expression was calculated as arbitrary expression units by a simplified ΔC_t_ method [38] normalizing the CK20- and EGFR expression against the reference gene TBP (TATA-box binding protein), as shown and further explained in Supplementary Table 1.

### Cell spiking experiments

The sensitivity of the CK20 qRT-PCR assay was determined by spiking of HT29 human colon cancer cells into fresh anti-coagulated blood of a healthy volunteer. HT29 cells were cultured in RPMI-1640 medium (Life Technologies) supplemented with 10% FBS (PAN-Biotech, Aidenbach, Germany), 1mM Glutamax and 1mM Na-pyruvate (Life Technologies). Total RNA from MNC fractions of blood samples spiked with 1000, 100, 10, and 1 HT29 cells per ml whole blood were analyzed by qRT-PCR as described above.

### Statistical analysis

Analyses were implemented for all subsets of clinical parameters in total and independently by tumor site and histopathological staging. Kaplan-Meier survival analyses were carried out for overall and disease-free survival (OS, DFS). For univariate analysis, significance was assessed by the log rank test. Dependence of the detection rate of biomarkers from clinical parameters was analyzed with the χ^2^ test after crosstab examination. Variables showing a significant association with the detection of a biomarker in univariate analysis, were included in multivariate models. Cox proportional hazard models were used in multivariate analysis. The area under the curve (AUC) of the Receiver-Operating-Characteristic (ROC) curve analysis was used to determine the prognostic value of CK20 mRNA expression. The Best-Youden-Index as the point of best sensitivity and specificity was calculated by ROC analysis and used to define the cut-off value.

All reported *P*-values are two-sided and were regarded statistically significant at ≤0.05. Statistical calculation and testing was performed with IBM SPSS Statistics 23.0 (IBM, München, Germany) and MedCalc (MedCalc Software, Ostend, Belgium)

## SUPPLEMENTARY MATERIALS FIGURES AND TABLE





## Abbreviations

AUC: area under the curve
CTC: circulating tumor cell
CTSC: circulating tumor stem cell
CK20: cytokeratin 20
CRC: colorectal carcinoma
DFS: disease free survival
DNA: desoxyribonucleic acid
EGFR: epidermal growth factor receptor
EpCAM: epithelial cell adhesion molecule
EU: expression units
HR: hazard ratio
MNC: mononuclear cells
mRNA: messenger ribonucleic acid
OS: overall survival
PBMC: peripheral blood mononuclear cells
PCR: polymerase chain reaction
RNA: ribonucleic acid
qRT-PCR: quantitative real-time polymerase chain reaction
ROC: receiver operating characteristic
UICC: Union internationale contre le cancer
