# Supplementary material for: Identifying patients with an unfavorable prognosis in early stages of colorectal carcinoma
Source: Oncotarget. 2018 Jun 8;9(44):27423–34. doi: 10.18632/oncotarget.25384 (PMC6007960; doi:10.18632/oncotarget.25384)
Supplement: Supplementary file 1 [file oncotarget-09-27423-s001.pdf]

Identifying patients with an unfavorable prognosis in early stages of colorectal carcinoma

SUPPLEMENTARY MATERIALS

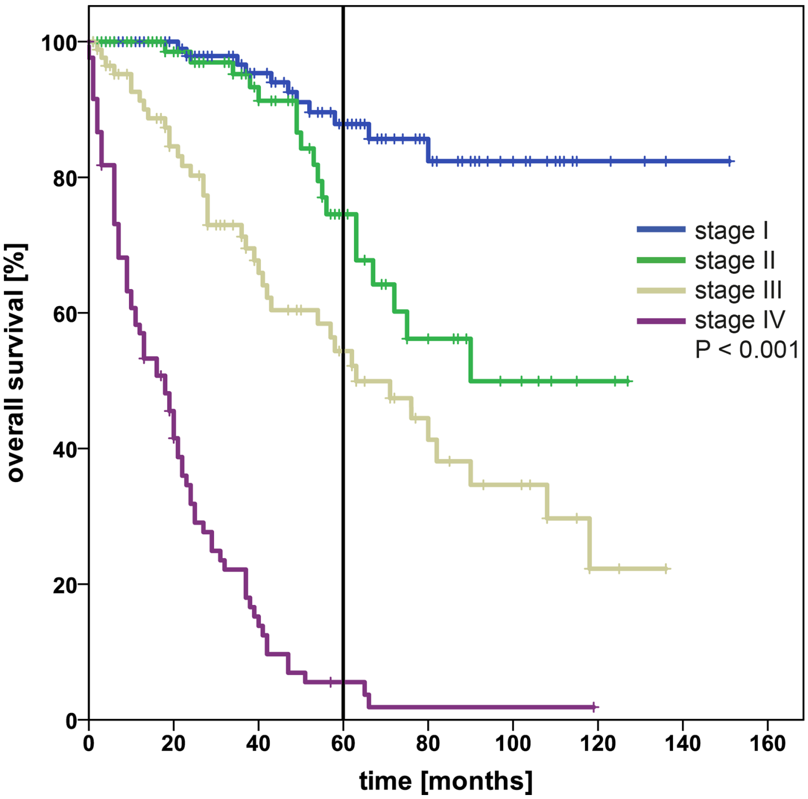

Supplementary Figure 1: Kaplan-Meier survival analysis of the cumulative overall survival of patients with colorectal carcinoma according to the UICC stage. The 5-year survival is indicated by the thick vertical line. The P-value was calculated by log-rank test.

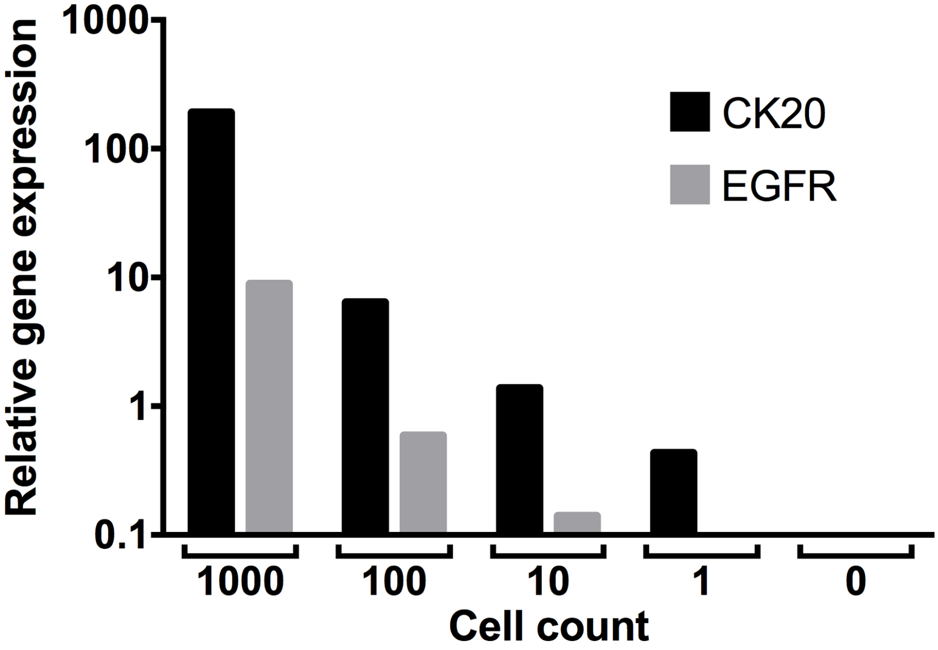

Supplementary Figure 2: Semi-quantitative real-time RT-PCR analysis of CK20 and EGFR expression in peripheral blood mononuclear cells fractions from healthy donor blood spiked with the indicated numbers per ml whole blood of HT29 colon adenocarcinoma cells. CK20: cytokeration 20; EGFR epidermal growth factor receptor.

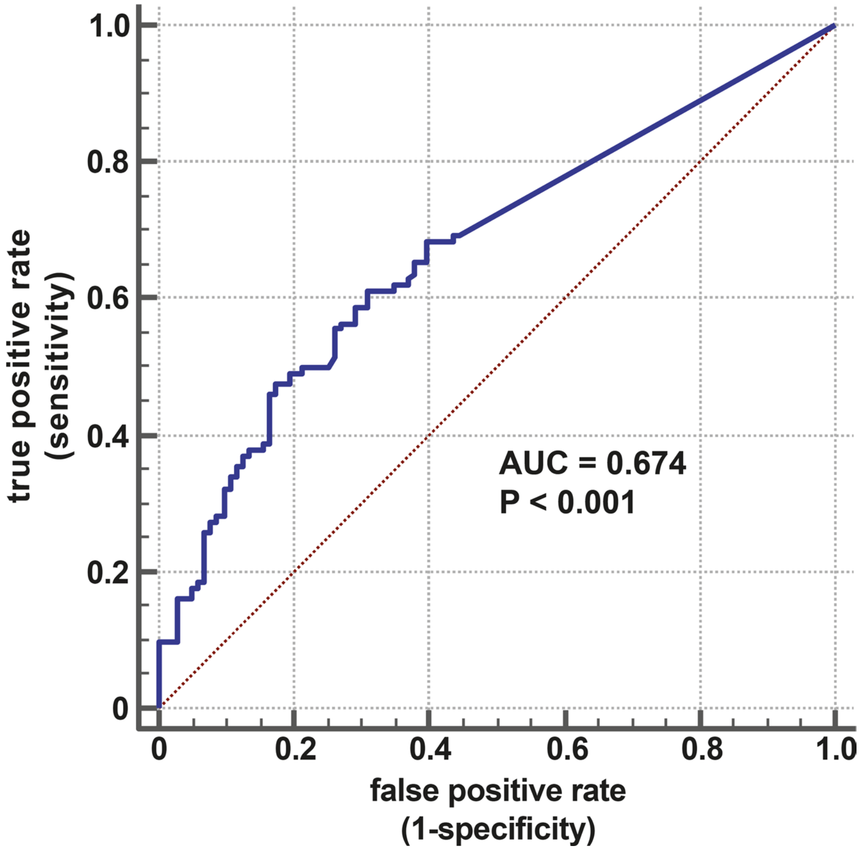

Supplementary Figure 3: Receiver-Operating Characteristic (ROC)-curve analysis of the prognostic value of the CK20 mRNA expression level with respect to the patients' survival; the Youden index was used to calculate a cut-off value for CK20 mRNA expression of 2.77 arbitrary expression units (EU). A significant relationship between CK20 expression above this cut-off and a shorter survival of the patients was demonstrated. AUC: area under the curve; CK20 cytokeratin 20.

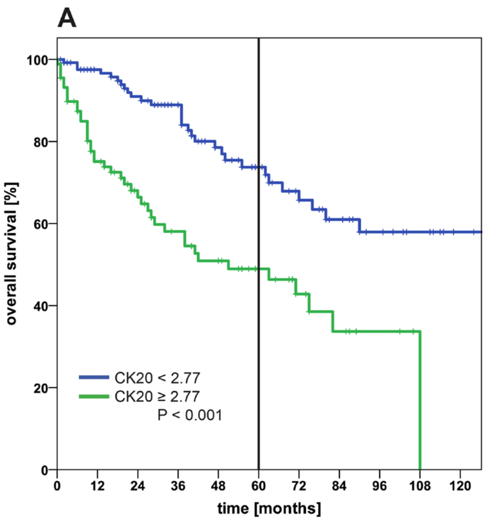

CK20 expression [EU]	patients at risk										
CK20 < 2.77	132	108	93	74	51	40	29	23	17	10	4
CK20 ≥ 2.77	90	60	41	33	27	19	11	6	3	0	0

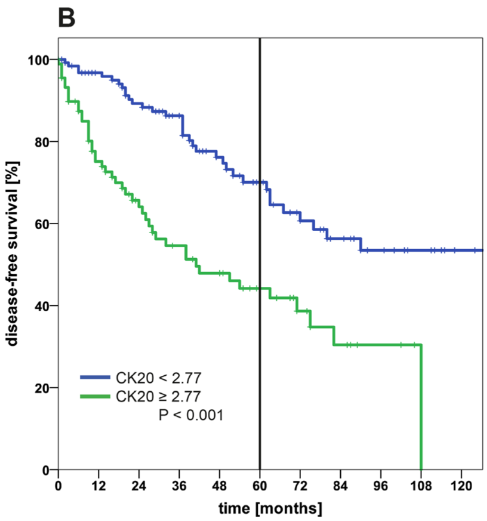

CK20 expression [EU]	patients at risk										
CK20 < 2.77	132	108	93	74	52	40	30	23	17	10	4
CK20 ≥ 2.77	90	60	41	33	27	19	11	7	3	0	0

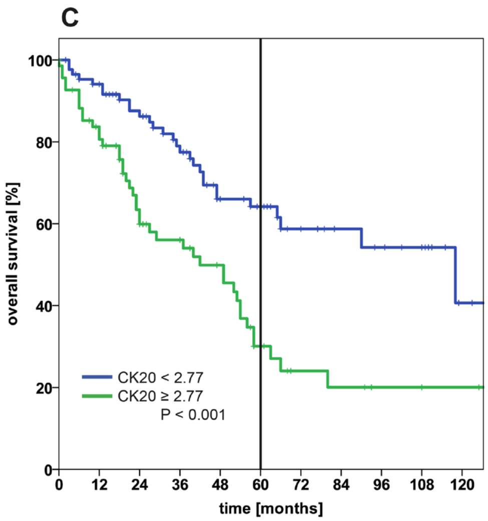

CK20 expression [EU]	patients at risk										
CK20 < 2.77	86	76	64	51	37	30	18	13	11	8	2
CK20 ≥ 2.77	69	53	35	28	23	11	6	4	3	2	2

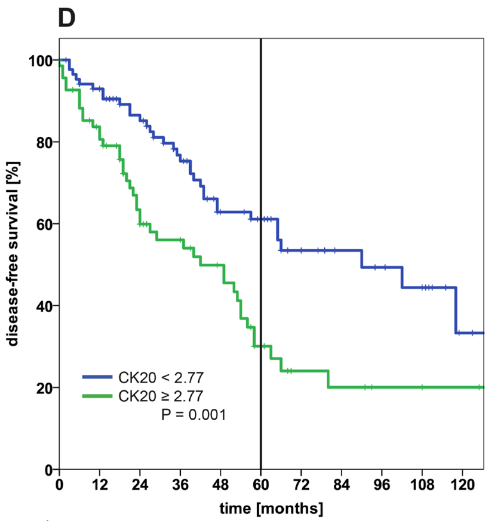

CK20 expression [EU]	patients at risk										
CK20 < 2.77	86	76	64	51	37	28	18	13	11	8	2
CK20 ≥ 2.77	69	53	35	28	23	11	6	4	3	2	2

Supplementary Figure 4: Kaplan-Meier survival analysis of the cumulative overall survival **(A, C)** and disease-free survival **(B, D)** according to the cytokeration 20 mRNA expression levels (high, ≥ 2.77 EU; low, < 2.77 EU). The tables under each plot show the number of patients at risk at each time point in the graph. the 5-year survival is indicated by the thick vertical lines. P-values were calculated by the log-rank test. CK20: cytokeratin 20; EU: expression units.

Supplementary Table 1: Quantitative real-time RT-PCR analysis of CK20 and EGFR expression in PBMC-fractions from venous blood of patients with colorectal adenocarcinoma

See Supplementary File 1
